# Comprehensive Analysis of JCHAIN as a Potential Prognostic Factor for Breast Cancer and an Indicator for Tumor Microenvironment

**DOI:** 10.3390/biomedicines13102366

**Published:** 2025-09-26

**Authors:** Yaqin Shi, Li Lin, Xinyu Zhu, Mengyao Wu, Caihua Xu, Wei Li, Kai Chen

**Affiliations:** 1 Department of Oncology, The First Affiliated Hospital of Soochow University, Suzhou 215000, China; shiyaqinand@126.com (Y.S.); mywu@suda.edu.cn (M.W.); caihuaxu@suda.edu.cn (C.X.); 2 Department of Radiology, The First Affiliated Hospital of Soochow University, Suzhou 215000, China; 15162725720@163.com; 3 Medical College, Soochow University, Suzhou 215000, China; 2330506091@stu.suda.edu.cn

**Keywords:** breast cancer, JCHAIN, tumor microenvironment, prognostic biomarker, immune cell, stromal component

## Abstract

**Background**: Breast cancer remains a predominant malignancy among females globally, and the tumor microenvironment (TME) exerts a pivotal role in its progression. Despite notable advancements in diagnostic and therapeutic modalities, resistance to conventional therapies persists as a critical hurdle, underscoring the necessity of exploring TME-related prognostic biomarkers. **Methods**: To elucidate the role of the TME in breast cancer progression and identify potential prognostic biomarkers, we analyzed RNA-seq data from 1081 breast cancer cases and 99 normal controls to assess tumor-infiltrating immune cells (TICs) and stromal components. Differential gene expression analysis identified genes correlated with ImmuneScore and StromalScore. A protein–protein interaction (PPI) network was constructed, followed by univariate Cox regression to pinpoint survival-associated genes. JCHAIN, significantly linked to survival outcomes, was selected for further investigation. Gene Set Enrichment Analysis (GSEA) and TIC correlation analyses were performed to explore its associations with immune pathways. Additionally, immunohistochemistry (IHC) and multiplexed immunofluorescence (mIF) were performed on 61 clinical samples. **Results**: High ImmuneScore was associated with improved survival. Joining chain of multimeric IgA and IgM (JCHAIN) expression was notably reduced in tumor tissues, with low expression correlating with poorer prognosis. GSEA highlighted immune-related pathways enriched in high JCHAIN expression groups. TIC analysis revealed positive correlations with CD8+ T cells and M1 macrophages. IHC and mIF validations further confirmed decreased JCHAIN protein expression in tumor tissues, and higher JCHAIN expression was associated with increased M1 macrophage density. **Conclusions**: JCHAIN serves as a promising prognostic biomarker in breast cancer, reflecting immune activity within the TME, providing valuable insights into immune-stromal interactions and the therapeutic potential of JCHAIN.

## 1. Background

Breast cancer arises as a common cancer in females worldwide, which is the leading cause of cancer-related death in women in 112 out of 183 countries worldwide in 2022 [1]. Despite significant advancements in diagnosis and treatment, the global incidence and mortality rates of breast cancer remain high [2]. Traditional treatment approaches primarily include chemotherapy, endocrine therapy, and HER-2 targeted therapy, but many patients eventually develop resistance. Therefore, there is a growing need for new therapeutic regimens and biomarkers to improve the management of breast cancer.

Breast cancer encompasses diverse biological types, each defined by unique pathological features, genetic profiles, and the tumor microenvironment (TME) [3]. TME in breast cancer plays a vital role in tumor characteristics and therapeutic response, and mainly comprises stromal cells, immune cells, cancer cells, and non-stromal factors. Since the TME is highly associated with treatment outcomes and genetic features, it is now increasingly recognized as a treatment target. Tumor-infiltrating lymphocyte (TIL), as the best characterized component of the TME, was highly associated with clinical outcomes. TIL cells encompass multiple groups of cells, including T cells, B cells, and NK cells [4]. The TIL population was more enriched in triple-negative subtypes compared with luminal tumors [5]. Regulatory T cells (Tregs) are more abundant in breast cancer than in normal breast tissues and are more relevant to high tumor grade, lymph node positivity, and poor prognosis [6]. Since the PD-1 inhibitor (pembrolizumab) combined with chemotherapy has been approved as standard treatment for advanced triple-negative breast cancer with positive PD-L1 expression (CPS ≥ 10), there has been an increasing trend to target TME-interacting molecules, which could potentially moderate the cytotoxic effect of immune cells [7]. Thus, the complexity of TME necessitates a deeper exploration of the interacting markers within the TME.

Transcriptome sequencing analysis has elucidated the role of various cell types in TME modulation. In this study, we utilized the ESTIMATE and CIBERSORT computational methods to determine the TIC proportion and the ratios of immune and stromal cell proportions of breast cancer samples from The Cancer Genome Atlas (TCGA) database. We indicated that joining the chain of multimeric IgA and IgM (JCHAIN) might act as a predictive biomarker.

## 2. Methods

### 2.1. Sample Database and Screening

Transcriptome RNA-seq profiles and clinical information of 1081 breast cancer samples and 99 normal samples were first downloaded from the TCGA database “https://portal.gdc.cancer.gov/ (accessed on 27 July 2024)”. The Cancer Genome Atlas Breast Carcinoma (TCGA-BRCA) dataset comprises multi-omics data from 1081 primary breast cancer patients, and includes whole exome sequencing, RNA sequencing, DNA sequencing, and protein array data. In addition, survival data were obtained from Xena clinical data [8].

### 2.2. Calculation of ImmuneScore, StromalScore and ESTIMATEScore

Using the ESTIMATE algorithm with R language and the Estimate package (v1.0.13) [9], we assessed the proportions of immune and stromal cells in the tumor microenvironment (TME) for each patient. The analysis produced three distinct scores: ImmuneScore, StromalScore, and ESTIMATEScore. Each score correlates with specific TME components: ImmuneScore and StromalScore reflect the relative abundance of immune cells and stromal cells, respectively, while ESTIMATEScore is a composite score derived from both components and serves as an indicator of tumor purity rather than a simple sum of cell abundances.

### 2.3. Survival Analysis and Clinical Information Analysis

Using the median values of ImmuneScore, StromalScore, and ESTIMATEScore, Kaplan–Meier survival curves were plotted with the ‘survival’ package (v3.7-0). The log-rank test was employed to assess statistical significance, with a *p*-value < 0.05 set as the criterion. In addition, the mRNA expression of JCHAIN and its relationship with prognosis were analyzed with ‘survival’ package (v3.7-0). A *p*-value < 0.05 was assessed as significant. The clinicopathological features of BRCA samples were downloaded from TCGA. Further analysis was performed with Prism 10.

### 2.4. Acquisition of DEGs from High-Score and Low-Score Groups Based on ImmuneScore and StromalScore

BRCA patient samples were sorted into high and low categories based on the median ImmuneScore and StromalScore, respectively. Differentially expressed genes (DEGs) between these two groups were identified using the package ‘limma’ (v3.40.2) in R. To account for multiple hypothesis testing, *p*-values were adjusted using the Benjamini–Hochberg (BH) method to calculate the false discovery rate (FDR). DEGs were defined as significant when meeting two criteria: an adjusted *p*-value (FDR) < 0.05 and a log fold change (FC) > 1. Heatmaps of the DEGs were generated by using R language with the pheatmap package. Additionally, GO and KEGG enrichment analysis was performed on the DEGs using R packages of enrichplot (v1.24.0), clusterProfiler [10], and ggplot2, with significant terms identified at *p* < 0.05.

### 2.5. PPI Network and COX Regression Analysis

The construction of the PPI network began with data from the STRING database, followed by a reconstruction process using Cytoscape version 3.6.2. For the network assembly, only nodes with an interaction confidence score exceeding 0.9 were selected. Univariate COX regression analysis was conducted using the survival package in R. The plot illustrates the top 17 genes, each with a *p*-value under 0.005, ordered from the smallest to the largest *p*-value. False Discovery Rate (FDR) method of Benjamini and Hochberg was used to adjust *p*-values for multiple comparisons.

### 2.6. Profiles of Tumor-Infiltrating Immune Cells

To estimate the TIC abundance profiles in all tumor samples, the CIBERSORT computational method was utilized. For BRCA samples, CIBERSORT was used to calculate TIC abundance, considering results with *p* < 0.05 for further examination. The comparison of TICs between high and low JCHAIN groups was conducted using the limma and vioplot packages. Furthermore, the relationship between JCHAIN and 21 different immune cell types was analyzed with the limma, ggplot2, ggpubr (v0.6.0) packages.

### 2.7. Obtaining Clinical Samples and Follow-Up Data

Tissue microarray containing ZL-Brcsur1221 was obtained from Punuoen Biotech (Nanjing, China), which contained cancer and adjacent specimens from 61 breast cancer patients. The patients underwent surgery, and the pathological results have been confirmed. The clinical staging was conducted according to the eighth edition of the TNM classification for breast cancer, as set by the American Joint Committee on Cancer (AJCC) and the Union for International Cancer Control (UICC). Follow-up data was collected through telephone interviews.

### 2.8. Gene Set Enrichment Analysis (GSEA)

Gene Set Enrichment Analysis was performed using R software (Version 4.4.0) with the ‘clusterProfiler’ and ‘enrichplot’ (v1.24.0) packages. A gene list was constructed using log2 fold change values from differentially expressed genes (DEGs), with gene symbols as names, and sorted in decreasing order. Gene sets were derived from Molecular Signatures Database (MSigDB) v7.0, including hallmark gene sets and immunologic gene sets.

### 2.9. Immunohistochemistry

Slides were deparaffinized using xylenes and ethanols. Antigen retrieval was then achieved through heat treatment with citrate buffer (BioGenex Laboratories, San Ramon, CA, USA). The specific antibodies against JCHAIN (ab269855) utilized are purchased from Abcam (Shanghai, China). Scanning of the immunostained sections was performed with a Zeiss microscope (Oberkochen, Germany). Immunohistochemical (IHC) Quantification Digital image analysis was performed using the Visiopharm Integrator System (Hørsholm, Denmark) with its Deep Learning AI-based analysis modules. For brightfield IHC staining (e.g., JCHAIN, Ki67), whole slide images were analyzed. The software was first trained to identify all nuclei to calculate the total cell count. Subsequently, an HDAB-DAB optical filter was applied to the region of interest (ROI) to segment cells based on staining intensity thresholds. Cells were automatically classified into four categories: negative (0), weak positive (1+), moderate positive (2+), and strong positive (3+). The H-Score was then automatically calculated by the software using the standard formula: H-Score = (1 × % 1+ cells) + (2 × % 2+ cells) + (3 × % 3+ cells).

### 2.10. Multiple Immunofluorescence and Quantitative Cell Phenotyping

Tissue microarray (TMA) sections from breast tumor samples were prepared for multiplexed immunofluorescence. After deparaffinization and antigen retrieval in citrate buffer (pH 6.0), non-specific binding was blocked with 5% normal serum. The sections were incubated overnight at 4 °C with a primary antibody panel against JCHAIN (ab269855; 1:8000; Abcam), CD163 (ab182422; 1:500; Abcam), CD68 (BX50031; 1:200; Bailin, Suzhou, China), and Pan Cytokeratin (Pan CK) (BX50143; 1:1000; Bailin). Following incubation with fluorophore-conjugated secondary antibodies, nuclei were counterstained with DAPI. The fluorophores were assigned as follows: orange for Pan CK, yellow for JCHAIN, green for CD163, and red for CD68. Slides were mounted with anti-fade mounting medium. Whole-slide imaging was performed using a high-resolution fluorescence slide scanner (Akoya Biosciences). For quantitative analysis, multi-component TIFF images were exported and analyzed with Visiopharm software (v2024.07) (Hørsholm, Denmark) using custom-built deep learning algorithms. The software was trained to perform nuclear detection (DAPI+) and multiplex phenotyping to automatically identify and quantify the following cell populations based on marker co-expression. The results were exported as cell densities (number of positive cells per mm^2^) for each phenotype within each TMA core for subsequent statistical comparison between patient groups.

### 2.11. Statistical Analysis

Data were analyzed and integrated with R software (Version 4.4.0) ”https://www.R-project.org (accessed on 27 July 2024)”. Statistical analysis was performed using Prism 9. Student’s unpaired t-test was applied for comparisons between two groups, while Kruskal–Wallis was used for multiple group comparisons; survival analysis methods (Kaplan–Meier curves, log-rank test, Cox proportional hazards regression) for assessing associations between variables and overall survival are detailed in Section 2.3. For descriptive and diagrammatic analysis of genes/cells: heatmaps for differential gene expression (via “pheatmap”), box/violin plots for gene expression/tumor-infiltrating immune cell proportions (via “ggplot2”/“vioplot”), scatter plots for correlations (via “ggplot2”/“ggpubr”), and GSEA enrichment plots (via “enrichplot”) were used, with specific implementations detailed in Section 2.4, Section 2.6 and Section 2.8. A *p*-value of less than 0.05 was considered statistically significant.

## 3. Results

Genetic RNA-seq data were obtained in 1081 breast cancer cases and 99 normal cases from the TCGA BRCA database. The shared differentially expressed genes (DEGs) from both the ImmuneScore and StromalScore were identified to build a protein interaction (PPI) network, which was also applied for further univariate COX regression analysis. This shared JCHAIN gene in the PPI network analysis and COX regression analysis was selected for further analysis. The JCHAIN gene has the most significant *p*-value in the univariate COX regression analysis. The association between JCHAIN expression and clinical features was evaluated in the TCGA BRCA database by using R language. In addition, the Gene Set Enrichment Analysis (GSEA) and TIC correlation analysis were applied to analyze the relations between JCHAIN expression and TME.

### 3.1. ImmuneScores Associated with the Prognosis of Breast Cancer Patients

**Figure 1:**
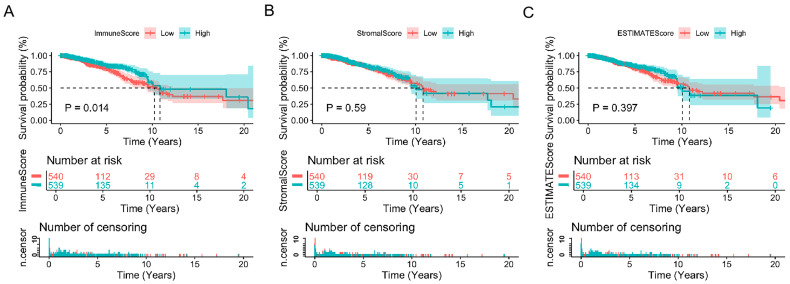
Impact of Scores and Breast Cancer Patient Survival: (**A**) Kaplan–Meier analysis comparing survival in high vs. low ImmuneScore groups, defined by the median (*p* = 0.014). (**B**) Survival curves for StromalScore, showing no significant difference (*p* = 0.59). (**C**) Kaplan–Meier survival analysis for ESTIMATEScore, with no significant difference (*p* = 0.397). Statistical significance between high- and low-score groups was assessed via the log-rank test.

To determine the correlation between the estimated score of immune and stromal components and survival, Kaplan–Meier survival curves were plotted with the ‘survival’ R package for ImmuneScore, StromalScore, and ESTIMATEScore. ImmuneScore and StromalScore represent the levels of immune and stromal components in TME, with higher scores indicating larger quantities. Summed by ImmuneScore and StromalScore, ESTIMATEScore reflects the overall components in the TME. The results demonstrated that the high ImmuneScore is positively correlated with improved overall survival rates (Figure 1A). However, the association between StromalScore and ESTIMATEScore and survival rate was not significant (Figure 1B,C), which indicates that the immune components within the TME are a better predictor of prognosis for breast cancer patients.

### 3.2. StromalScores Correlated with Clinicopathological Stages in Breast Cancer Patients

**Figure 2:**
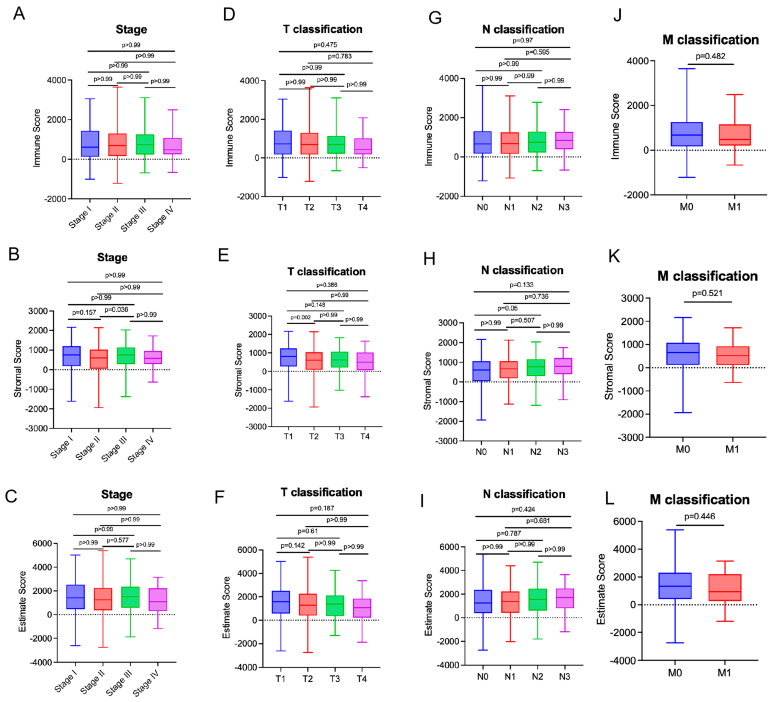
Relationship of ImmuneScore and StromalScore with Clinicopathological Characteristics: (**A**–**C**) Results of Kruskal–Wallis rank sum test for ImmuneScore, StromalScore, and ESTIMATEScore across different cancer stages. (**D**–**F**) Distribution of scores across T classifications, analyzed using the Kruskal–Wallis rank sum test. (**G**–**I**) Comparison of the scores in N classifications via the Kruskal–Wallis rank sum test. (**J**–**L**) Results from Wilcoxon rank sum test for ImmuneScore, StromalScore, and ESTIMATEScore in M classification.

Furthermore, we analyzed the breast cancer clinical data from the TCGA database to investigate whether the immune and stromal component proportions are related to clinicopathological characteristics. The results reveal that StromalScore is statistically different between stage Ⅱ and Ⅲ patients (Figure 2A–C, *p* = 0.036). In addition, the stromal score is more elevated in T1 patients compared with T2 patients (Figure 2D–F, *p* = 0.002). In addition, both EstimateScore and ImmuneScore showed no correlation with clinical staging or TNM staging in breast cancer (Figure 2). These results suggest a link between stromal components and breast cancer progression, including aspects such as invasion and metastasis.

### 3.3. Enrichment of Immune-Related DEGs at the Intersection of ImmuneScore and StromalScore

**Figure 3:**
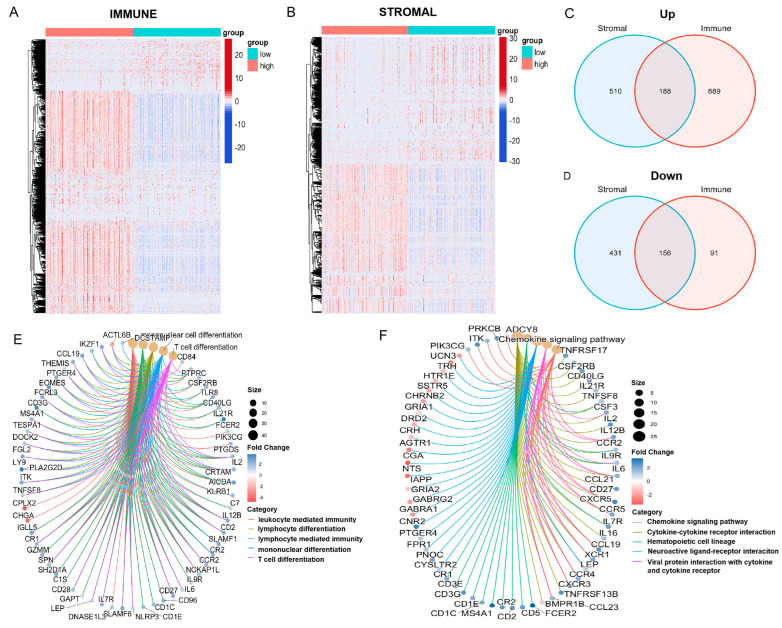
Differential Gene Expression Analysis Based on Stromal and ImmuneScores in Breast Cancer: (**A**,**B**) Heatmaps illustrating differentially expressed genes between high and low StromalScore (**A**) and ImmuneScore (**B**) groups. (**C**,**D**) Venn diagrams depicting the intersection of differentially expressed genes in the StromalScore and ImmuneScore groups. (**C**) For upregulated genes, 188 are shared between two groups, while 510 are unique to StromalScore and 689 to ImmuneScore. (**D**) For downregulated genes, 156 are common to both, with 431 exclusive to StromalScore and 91 to ImmuneScore. (**E**,**F**) GO and KEGG pathway enrichment analysis of 344 DEGs, with a significance threshold of *p* < 0.05 for enrichment.

In order to identify gene profile changes in TME related to immune and stromal components, we compared the gene expression between the high- and low-score samples. The analysis of ImmuneScore revealed 1124 DEGs, with 877 up-regulated and 247 down-regulated genes (Figure 3A). In addition, the analysis of StromalScore identified 1285 differentially expressed genes (DEGs), with 698 up-regulated and 587 down-regulated (Figure 3B). The Venn diagram showed 188 up-regulated genes that are shared by the high ImmuneScore and StromalScore samples, and 156 down-regulated genes shared by the low ImmuneScore and StromalScore cases, for a total of 344 DEGs (Figure 3C,D). Further GO enrichment analysis illustrated that these DEGs were involved in immune-related functions, such as leukocyte mediated immunity, lymphocyte differentiation, lymphocyte mediated immunity, mononuclear differentiation, and T cell differentiation processes (Figure 3E). In addition, KEGG analysis highlighted key processes including chemokine signaling pathways, cytokine-cytokine receptor interaction, hematopoietic cell lineage, neuroactive ligand–receptor interaction and viral protein interaction with cytokine and cytokine receptor (Figure 3F). Therefore, these DEGs were highly related to immune functions, suggesting that immune factors are a major feature of the TME in breast cancer.

### 3.4. Critical Genes Associated with Pathophysiological Mechanisms and Survival Outcome

**Figure 4:**
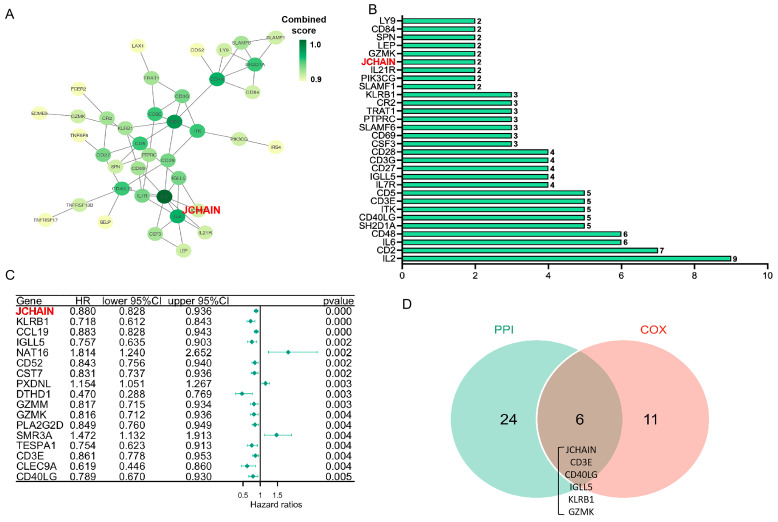
Key Gene Identification and Prognostic Significance: (**A**) Protein–protein interaction (PPI) network of differentially expressed genes, with an interaction confidence threshold > 0.9. Darker colors represent higher confidence in interactions. (**B**) Bar plot of the top 30 genes from the PPI network, ranked by interaction confidence scores. (**C**) Forest plot of Cox proportional hazards regression analysis for 344 genes, highlighting the most significant factors with *p* < 0.005. (**D**) Venn diagram illustrating the overlap between genes in the PPI network (green) and those with significantly prognostic value in the Cox regression analysis (red).

To identify critical genes involved in both pathophysiological mechanisms and survival outcomes, we constructed a PPI network based on the STRING database using Cytoscape software version 3.6.2 (Figure 4A). The top 30 genes were selected for further analysis (Figure 4B). The univariate COX regression analysis was conducted to determine which genes were significantly associated with survival outcomes (Figure 4C). The top 17 genes ranked by the *p*-value of univariate COX regression were identified. Among the top 30 genes in the PPI construction, JCHAIN exhibits the most significant *p*-value associated with survival prognosis (Figure 4D). JCHAIN, named as the joining chain of multimeric IgA and IgM, enables IgA binding activity and protein homodimerization activity. JCHAIN was identified as a critical gene involved in both pathophysiological mechanisms and survival outcomes [11]. This finding suggests that JCHAIN may play a central role in breast cancer development and patient survival.

### 3.5. The Characteristics of JCHAIN mRNA Expression in Breast Cancer and Its Relationship with Clinical Indicators and Prognosis

**Figure 5:**
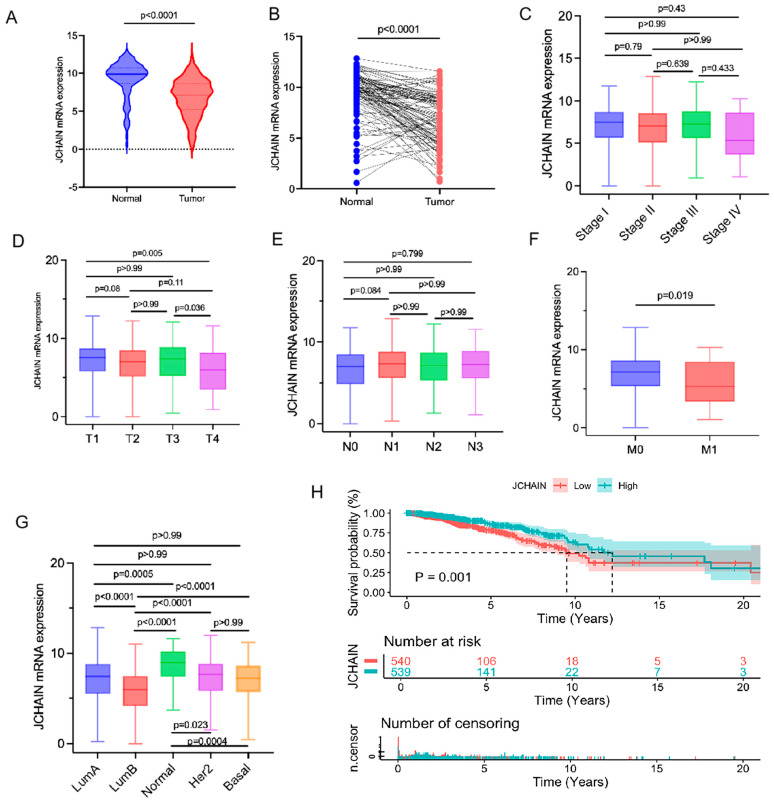
JCHAIN expression in Normal vs. Tumor tissues and its Clinical Impact: (**A**) Violin plot comparing JCHAIN mRNA expression in normal and tumor tissues (*p* < 0.0001). Statistical comparisons were performed using the Wilcoxon rank sum test. (**B**) Paired analysis of JCHAIN mRNA expression in normal and tumor samples, showing significant upregulation in tumors (*p* < 0.0001). (**C**–**F**) Box plots comparing JCHAIN mRNA expression across tumor stages, T stages (T1–T4) (*p* < 0.01), N stages (N0–N3), and M stages (M0 vs. M1) (*p* < 0.05). Wilcoxon rank sum or Kruskal–Wallis rank sum test served as the statistical significance test. (**G**) JCHAIN mRNA expression across different breast cancer subtypes (LumA, LumB, Her2, Basal) vs. normal tissues by Kruskal–Wallis rank sum test. (**H**) Kaplan–Meier survival analysis of patients showing that high JCHAIN expression is associated with better prognosis (*p* = 0.001). Significance was assessed via the log-rank test. All the above data are derived from the TCGA database.

**Figure 6:**
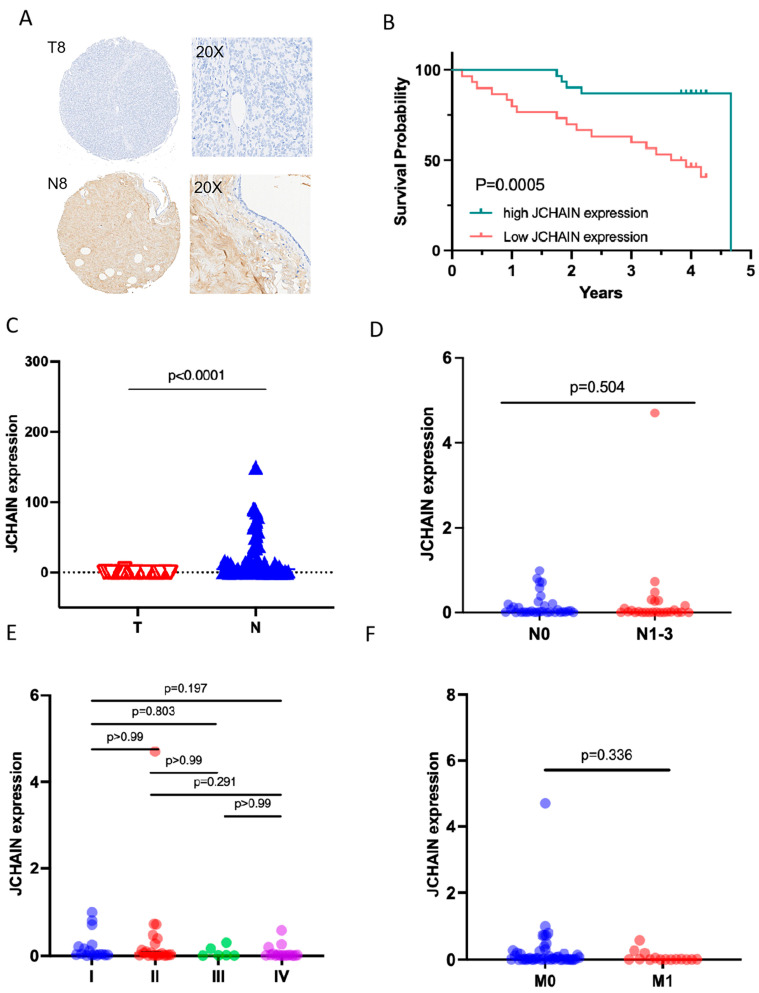
JCHAIN expression and prognostic significance of JCHAIN in the 61 pairs of clinical breast cancer tissues: (**A**) Representative immunohistochemical staining of JCHAIN in tumor tissues. (**B**) Kaplan–Meier survival curves comparing overall survival between patients with high vs. low JCHAIN expression. (**C**) Unpaired Student’s test comparing JCHAIN expressions between tumor and normal tissues. (**D**–**F**) Analysis of the association between JCHAIN expression and clinical parameters, including stages I–IV, lymph node status, metastasis status. Statistical comparisons were analyzed via the Kruskal–Wallis rank sum test.

JCHAIN mRNA expression was compared between tumor and normal samples in breast cancer. As shown in Figure 5A, tumor tissues exhibited significantly lower JCHAIN mRNA levels, a trend also observed in paired tumor-normal tissues comparisons, where tumor tissues and their corresponding normal adjacent tissues were obtained from the same patients (Figure 5B). Further analysis across different stages (I–IV) and classifications (T1–T4 for tumor size, NO–N3 for lymph node involvement, and M0- M1 for metastasis) indicated a significant correlation between JCHAIN mRNA expression and T/M classifications in TMN staging (*p* < 0.05) (Figure 5C–F). However, no notable correlation was found with clinical staging or N classification (Figure 5C,E). Subtype analysis showed that JCHAIN mRNA expression varied significantly, with the lowest levels in the luminal B subtype (Figure 5G). Survival analysis indicated that reduced JCHAIN mRNA expression was relevant to poorer survival outcomes. In addition, we evaluated the JCHAIN expression and its prognostic role in the 61 pairs of clinical breast cancer tissues (Figure 6). JCHAIN protein expression was decreased in breast tumors compared to normal tissues (Figure 6A,C). Kaplan–Meier analysis revealed that low JCHAIN expression was associated with shorter overall survival (Figure 6B). However, no significant association was found between JCHAIN expression and clinical stage, lymph node status, and metastasis status (Figure 6D–F), possibly due to the limited sample size. These findings suggest that decreased JCHAIN expression may be associated with tumor progression and worse prognosis, highlighting its potential as a prognostic marker. All the above data are derived from the TCGA database.

### 3.6. JCHAIN Holds the Potential to Be an Indicator of TME Modulation

**Figure 7:**
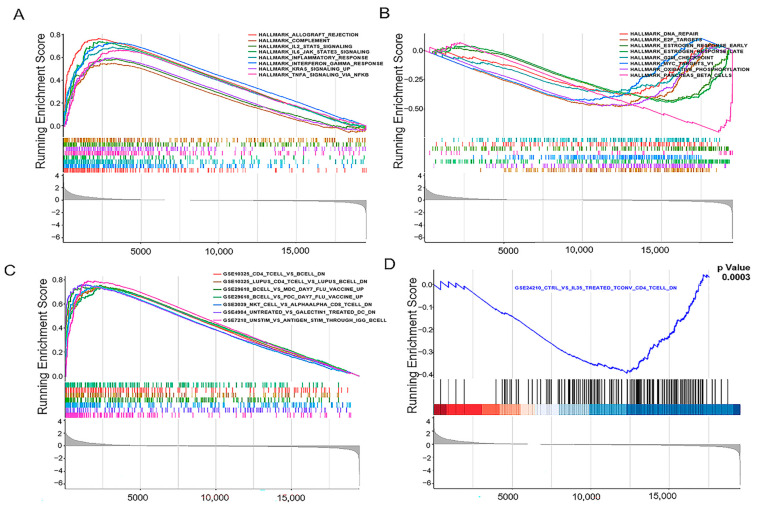
Gene Set Enrichment Analysis (GSEA) for patients with high and low JCHAIN expression: (**A**) GSEA of hallmark gene sets in the high JCHAIN expression group, highlighting immune and inflammatory pathways, including complement, IL6-JAK-STAT3, inflammatory response, Interferon gamma signaling, KRAS signaling, and TNFA signaling via NF-kB. Distinct enrichment patterns are shown in the enrichment score (ES) curves. (**B**) GSEA in the low JCHAIN expression group, enriched in estrogen responses, cell cycle, and transcriptional regulation pathways. (**C**) Immune-related functional gene sets enriched in the high JCHAIN expression group in C7 collection. (**D**) GSEAfor the iSE2410 dataset (CTRL vs IL35-treated TCONV CD4 T cells),showing significant enrichment with a *p*-value of 0.0003. In subfigure (**D**), the black line marks gene positions, and the red-blue heatmap shows expression abundance (deeper red = larger logFC, deeper blue = smaller logFC).

Since JCHAIN levels are negatively correlated with both survival and T/M classifications in the TMN stages of BRCA patients, GSEA was carried out in both the high and low expression groups, respectively. The GSEA plots illustrate the enrichment of biological pathways associated with JCHAIN expression (Figure 7A). The running enrichment score along the x-axis and the gene set positions on the y-axis were presented. In the high JCHAIN expression group, immune response pathways were prominently enriched, including complement activation, IL2 STAT5 signaling, IL6 JAK STAT3 signaling, and inflammatory response (Figure S1A). On the other hand, genes in the low JCHAIN expression group were primarily enriched in pathways such as hallmark E2F targets, early and late estrogen response, and the G2M checkpoint signaling (Figure 7B). In the C7 collection defined by MSigDB, which comprises the immunologic gene sets, numerous immune-related functional gene sets were enriched in the high JCHAIN mRNA expression group (Figure 7C). However, few gene sets were enriched in the low JCHAIN expression group (Figure 7D). These findings indicated that JCHAIN could serve as a potential biomarker for assessing the status of TME.

### 3.7. Correlation of JCHAIN with the Proportion of TICs

**Figure 8:**
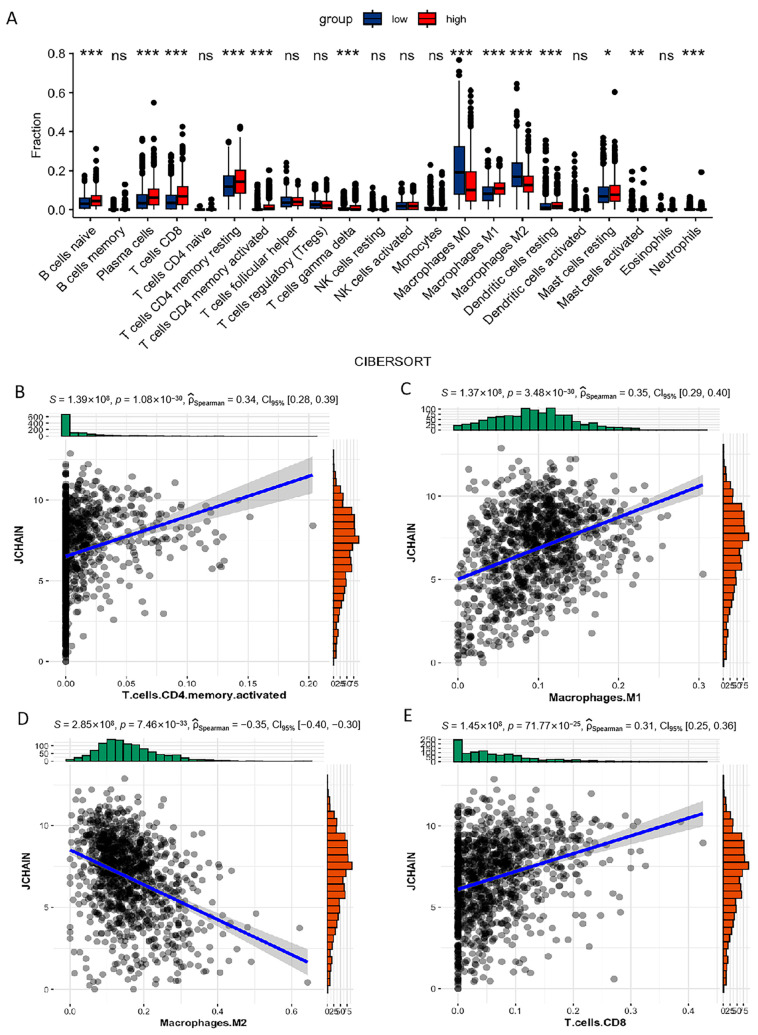
Immune cell fraction and correlations with JCHAIN expression: (**A**) Boxplot showing the immune cell fractions from CIBERSORT in low vs. high JCHAIN expression groups, and Wilcoxon rank sum was used for the significance test, where * indicates *p* < 0.05, ** indicates *p* < 0.01, *** indicates *p* < 0.001. (**B**–**E**) Scatter plots showing correlations between JCHAIN expression and specific immune cell fractions: (**B**) T cells CD4 memory activated, (**C**) Macrophages M1, (**D**) Macrophages M2, and (**E**) T cells CD8. Spearman correlation coefficients (ρ), *p*-values, and 95% confidence intervals are displayed for each plot.

To further explore the relationship between JCHAIN expression and the immune microenvironment, a profile of 22 immune cell types in BRCA samples was constructed. The CIBERSORT computational method was applied to estimate the abundance of TICs and assess their correlation with JCHAIN expression (Figure S1A,B). As shown in Figure 8, some TICs showed a positive correlation with JCHAIN. For instance, plasma cells and CD4 T memory activated cells also displayed a positive correlation with JCHAIN (Figure 8A,B). Additionally, CD8 T cells exhibited a tendency to increase in proportion as the expression level of JCHAIN increased (Figure 8E). M1 Macrophages showed an elevation in proportion as JCHAIN expression increased, and a negative correlation was observed in M2 macrophage cells (Figure 8C,D). Significance testing was performed using the Wilcoxon rank sum test, and further study on the correlation between 4 TICs and JCHAIN expression (*p* < 0.05) was conducted. Each plot included a blue line representing a fitted linear model, indicating the immune cell proportion trends along with JCHAIN expression. Spearman correlation coefficients were used for the correlation test.

**Figure 9:**
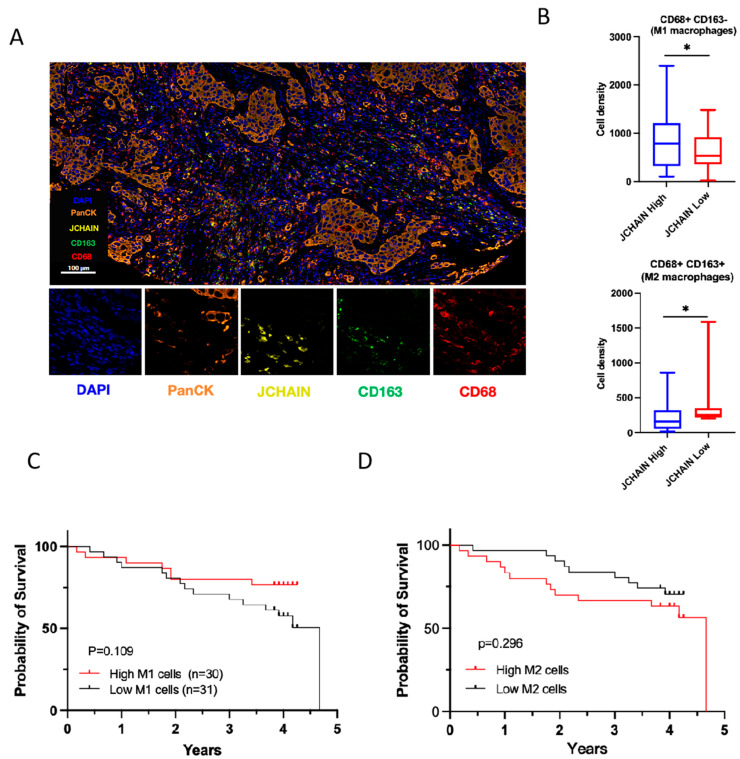
Multiplexed quantitative immunofluorescence analysis of the tumor microenvironment with cell markers: (**A**) Representative staining of cells: DAPI (blue)/PanCK (orange)/JCHAIN (yellow)/CD163 (green)/CD68 (red). The scale bar represents 100 µm. (**B**) Box plots showing the density of macrophages in relation to JCHAIN expression. Statistical comparison was performed using the Wilcoxon rank sum tes, where * indicates *p* < 0.05. (Top) M1 macrophage density (CD68+ CD163−) is higher in tumors with high JCHAIN expression. (Bottom) M2 macrophages (CD68+ CD163+) show a significant increase in cell density in tumors with low JCHAIN expression. (**C**) Kaplan–Meier survival curve for M1 macrophage density. Patients were divided into high (n = 30) and low (n = 31) M2 macrophage groups. No significant difference between high and low M1 cell groups (*p* = 0.109). (**D**) Kaplan–Meier survival analysis based on M2 macrophage density. Although the high M2 group showed a trend toward poorer overall survival, the difference did not reach statistical significance according to the log-rank test (*p* = 0.296).

We further conducted the multiplexed quantitative immunofluorescence analysis to analyze the M1/M2 macrophage patterns in the tumor microenvironment (Figure 9A). M1 macrophage density (CD68+ CD163−) was higher in tumors with high JCHAIN expression, while M2 macrophages (CD68+ CD163+) were more abundant in tumors with low JCHAIN expression (Figure 9B). Kaplan–Meier survival analysis showed no significant survival difference for high vs. low M1 macrophage density (*p* = 0.109). For the patients with higher M2 macrophage infiltration, there is a worse survival trend compared with those with lower infiltration. However, the log-rank test indicated no statistically significant difference between the two groups (*p* = 0.296). These findings suggest that, while M2 macrophages may contribute to unfavorable prognosis, the current dataset lacks sufficient power to establish statistical significance. Larger cohorts or integrative analyses considering other immune-related markers, such as the M1/M2 ratio, may be required to further clarify the prognostic role of M2 macrophages.

## 4. Discussion

This study explored the role of JCHAIN in breast cancer, with a focus on its relationship to the tumor microenvironment (TME) and its prognostic significance. By examining the ImmuneScore, StromalScore, and ESTIMATEScore in breast cancer patients, we gained meaningful insights into the dynamic nature of the TME. The TME functions as a complex and dynamic ecosystem, where the ImmuneScore and StromalScore serve as key indicators of its composition. A high ImmuneScore was positively associated with improved overall survival, supporting the established understanding that a strong immune response within the TME plays a vital role in limiting tumor growth and metastasis [12]. Elevated ImmuneScores likely reflect a favorable immune-tumor balance, resulting in better patient outcomes.

The StromalScore’s associations with clinical stages and T/N classifications indicate that stromal components are involved in breast cancer progression. Specifically, its negative correlation with clinical stages and T classification suggests that higher stromal content may correspond to a more advanced disease state and greater tumor invasiveness. Through comparisons of the gene expression profiles between high- and low-score samples based on ImmuneScore and StromalScore, we identified differentially expressed genes (DEGs). Subsequent GO and KEGG enrichment analysis provided insights into the biological functions and pathways of these DEGs. Additionally, we constructed a protein–protein interaction (PPI) network and performed univariate COX regression analysis, highlighting JCHAIN as a significant candidate gene. These findings suggest JCHAIN’s potential as a key factor in the underlying biological and pathophysiological mechanisms of breast cancer and its impact on survival outcomes.

The discovery of JCHAIN as a critical gene with prognostic value is a central finding of our study. The consistently lower JCHAIN mRNA expression in tumor samples compared to normal samples, in conjunction with its association with TNM staging and breast cancer subtypes, strongly suggests that JCHAIN is intricately involved in the biological processes that drive breast cancer progression. In the context of the TME, the reduced JCHAIN expression may disrupt the normal balance between the tumor and the immune/stromal components. Notably, the lowest expression in the luminal B subtype may imply a subtype-specific role for JCHAIN, potentially contributing to the relatively poorer prognosis observed in this subtype compared to others. Survival analysis has further validated the prognostic importance of JCHAIN, with lower mRNA expression being a reliable predictor of poor survival outcomes. This finding is in line with the hypothesis that JCHAIN may be involved in promoting tumor growth, invasion, and metastasis. JCHAIN could regulate genes and pathways that are essential for cancer cell survival and proliferation. Its downregulation could lead to a more aggressive tumor phenotype with increased metastatic potential and decreased patient survival.

To analyze the additional functional insights into JCHAIN’s role within the TME, we conducted GSEA. The enrichment of immune response pathways in the high JCHAIN expression group suggests that JCHAIN may be involved in modulating the immune response within the TME. It could potentially enhance the activation and function of immune cells, thereby creating a more favorable immune microenvironment for cancer control. JCHAIN might act as a signaling molecule that activates immune cells or promotes their recruitment to the tumor site. In contrast, the enrichment of different pathways in the low-expression group of JCHAIN suggests that JCHAIN may have a complex regulatory role in the cell cycle within breast cancer biology [13,14,15]. Meanwhile, in the C7 immune gene set collection, there are relatively few immune-related functional gene sets enriched in the low-expression group of JCHAIN. This indicates that in the low-expression state, JCHAIN may not be able to effectively participate in or regulate immune-related biological processes. As a result, the functional activation of immune cells and the coordination of immune responses may be inhibited. This makes tumor cells more likely to escape immune surveillance and attacks, thereby facilitating the survival, proliferation, and metastasis of tumor cells in the body and ultimately having an adverse impact on the prognosis of patients. Subsequently, we conducted an analysis of the correlation between the expression of JCHAIN and the proportion of TICs. This analysis further elucidates the role of JCHAIN in shaping the immune microenvironment. The positive correlation with certain TICs, such as memory B cells and plasma cells, and the negative correlation with others, like M1 Macrophages and activated NK cells, suggest that JCHAIN may influence the delicate balance between anti-tumor and pro-tumor immune responses. This balance is extremely crucial for tumor development [16]. When the expression of JCHAIN is abnormal, it might break the original immune balance and create a microenvironment that is either favorable for tumor growth or inhibitory to it.

Notably, JCHAIN exhibits distinct dysregulation patterns and prognostic roles across malignancies, with its prognostic significance in breast cancer showing both overlaps and contrasts with other tumor types. In intrahepatic cholangiocarcinoma (ICC), JCHAIN is a core marker of the “plasma cell+” immune infiltration pattern (co-expressed with IGHG1), which correlates with favorable overall survival by enhancing anti-tumor immunity. By contrast, ICC’s “MARCO+ tumor-associated macrophage (TAM)” pattern is accompanied by JCHAIN downregulation and suppresses T-cell infiltration [17]. In adult Hispanic B-cell acute lymphoblastic leukemia (B-ALL), high JCHAIN expression-part of a 3-gene signature with ID1 and ID3-predicts poor induction therapy response and shortened survival, driven by NF-κB pathway hyperactivation [18].

Mechanistically, JCHAIN downregulation in breast cancer involves three interconnected pathways. First, oncogenic NF-κB signaling transcriptionally represses JCHAIN—prioritizing pro-inflammatory cytokine expression over JCHAIN while promoting epithelial–mesenchymal transition (EMT) [19]. Second, JCHAIN is essential for disrupting IgM pentamer symmetry. Its loss impairs IgM assembly and disrupts B-T cell crosstalk [20]. Third, the tumor microenvironment (TME) contributes to JCHAIN suppression.

While this study provides novel insights into JCHAIN’s role in breast cancer, several limitations warrant consideration. First, transcriptomic data from TCGA may not fully recapitulate protein-level expression patterns, as mRNA abundance does not always correlate with functional protein activity. Second, further functional validation (e.g., in vitro/in vivo mechanistic studies) is required to establish causal relationships between JCHAIN and TME status. Finally, immunohistochemistry validation in 61 clinical samples restricts subgroup analysis power for clinicopathological features like metastasis, necessitating replication in larger cohorts. These limitations highlight the need for multi-omics integration and mechanistic studies to advance JCHAIN’s translational potential.

## 5. Conclusions

This study identifies JCHAIN as a novel prognostic biomarker and indicator reflecting immune activity in the tumor microenvironment (TME) in breast cancer. Integrated bioinformatics and clinical validation revealed that reduced JCHAIN expression correlates with aggressive tumor phenotypes, immunosuppressive TME signatures, and poor survival. Mechanistically, JCHAIN upregulation was associated with immune activation pathways and cytotoxic immune cell infiltration, whereas downregulation promoted cell cycle progression and epithelial–mesenchymal transition. JCHAIN was identified as a critical mediator of immune-stromal interactions, particularly in luminal B and triple-negative breast cancer subtypes. LumB subtype exhibited the lowest JCHAIN mRNA expression, and low JCHAIN in this subtype correlated with poor survival—consistent with its function in sustaining immune activation pathways and cytotoxic immune cell infiltration.

In general, our research undertakes a comprehensive analysis of JCHAIN as a potential prognostic factor for breast cancer and an indicator of tumor microenvironment reconstruction. It offers a new research direction and target for a deeper understanding of the pathogenesis of breast cancer, the development of new treatment strategies, and the improvement of patient prognosis.

## Data Availability

The original data presented in the study are openly available in the TCGA database at https://portal.gdc.cancer.gov/ (accessed on 27 July 2024). The other data presented in this study are available on request from the corresponding author due to privacy, legal and ethical reasons.

## Supplementary Materials

The following supporting information can be downloaded at: https://www.mdpi.com/article/10.3390/biomedicines13102366/s1, Figure S1: Immune cell composition and correlations.

## Abbreviations

AJCC	American Joint Committee on Cancer
B-ALL	B-cell Acute Lymphoblastic Leukemia
CAFs	Cancer-Associated Fibroblasts
CD	Cluster of Differentiation
DEGs	Differentially Expressed Genes
DAPI	4′,6-Diamidino-2-Phenylindole
EMT	Epithelial–Mesenchymal Transition
ER	Estrogen Receptor
GSEA	Gene Set Enrichment Analysis
HER-2	Human Epidermal Growth Factor Receptor 2
ICC	Intrahepatic Cholangiocarcinoma
IHC	Immunohistochemistry
IL	Interleukin
JAK	Janus Kinase
JCHAIN	Joining Chain of Multimeric IgA and IgM
GO	Gene Ontology
KEGG	Kyoto Encyclopedia of Genes and Genomes
mIF	Multiplex Immunofluorescence
PD-1	Programmed Cell Death Protein 1
PD-L1	Programmed Cell Death Ligand 1
PPI	Protein–Protein Interaction
STAT	Signal Transducer and Activator of Transcription
TAM	Tumor-Associated Macrophage
TCGA	The Cancer Genome Atlas
TICs	Tumor-Infiltrating Immune Cells
TME	Tumor Microenvironment
TNBC	Triple-Negative Breast Cancer
TNM	Tumor-Node-Metastasis
UICC	Union for International Cancer Control
