# Supplementary material for: Comprehensive Analysis of JCHAIN as a Potential Prognostic Factor for Breast Cancer and an Indicator for Tumor Microenvironment
Source: Biomedicines. 2025 Sep 26;13(10):2366. doi: 10.3390/biomedicines13102366 (PMC12561060; doi:10.3390/biomedicines13102366)
Supplement: Supplementary file 1 [file biomedicines-13-02366-s001.zip › biomedicines-3787563-supplementary.pdf]

Supplementary File:

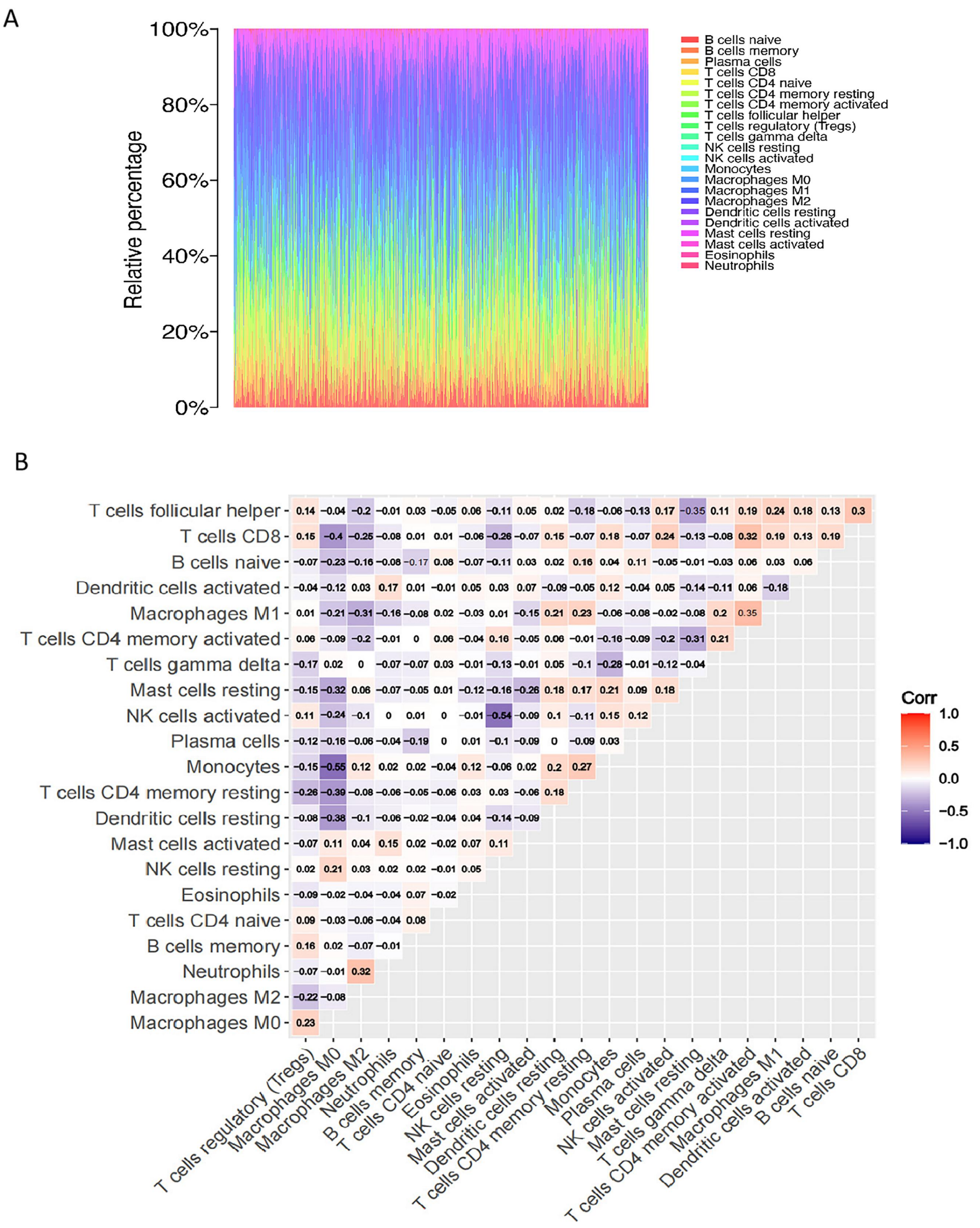

Figure S1. Immune cell composition and correlations. (A) Barplot showing the relative percentages of different immune cell types. Each cell type is color-coded as indicated. (B) Correlation heatmap of various immune cell types. The color intensity represents the strength of the correlation, with red indicating a positive correlation and blue indicating a negative correlation.
